# Non-coding RNAs in Pancreatic Ductal Adenocarcinoma

**DOI:** 10.3389/fonc.2020.00309

**Published:** 2020-03-17

**Authors:** Ruining Gong, Yueping Jiang

**Affiliations:** Department of Gastroenterology, Affiliated Hospital of Qingdao University, Qingdao, China

**Keywords:** non-coding RNAs, pancreatic ductal adenocarcinoma, miRNA, lncRNA, circ-RNA

## Abstract

Non-coding RNAs (ncRNAs) are reported to be expressed in human cancers, including pancreatic ductal adenocarcinoma (PDAC). These ncRNAs affect the growth, migration and invasion of tumor cells by regulating cell cycle and apoptosis, as well as playing important roles in epigenetic processes, transcription and post-transcriptional regulation. It is still unclear whether alterations in ncRNAs influence PDAC development and progression. Because of this, analysis based on existing data on ncRNAs, which are crucial for modulating pancreatic tumorigenesis, will be important for future research on PDAC. Here, we summarize ncRNAs with tumor-promoting functions: HOTAIR, HOTTIP, MALAT1, lncRNA H19, lncRNA PVT1, circ-RNA ciRS-7, circ-0030235, circ-RNA_100782, circ-LDLRAD3, circ-0007534, circRHOT1, circZMYM2, circ-IARS, circ-RNA PDE8A, miR-21, miR-155, miR-221/222, miR-196b, miR-10a. While others including GAS5, MEG3, and lncRNA ENST00000480739, has_circ_0001649, miR-34a, miR-100, miR-217, miR-143 inhibit the proliferation and invasion of PDAC. Hence, we summarize the functions of ncRNAs in the occurrence, development and metastasis of PDAC, with the goal to provide guidance in the clinical diagnosis and treatment of PDAC.

## Introduction

Pancreatic ductal adenocarcinoma (PDAC) is a malignant tumor with a high incidence, malignancy and mortality rate. PDAC is the seventh leading cause of cancer-related death throughout the world (1). According to 2019 statistics from the American Cancer Society, PDAC mortality rate ranks 4th in both men and women (2). Due to a lack of effective treatments, the 5-years survival rate for PDAC remains below 8% (3). This high mortality rate is largely due to late presentation and detection of the disease, when patients become non-candidates for surgical resection. In addition, the mechanisms behind PDAC tumorigenesis and progression are still unclear.

Mutations in *KRAS, TP53, SMAD4, CDKN2A* commonly contribute to PDAC progression (4–7). In addition, PDAC development requires the involvement of various signal transduction pathways including the Hippo, Hedgehog, Wnt/Notch, JNK, PI3K, K-ras, and transforming growth factor (TGF) -β signaling pathways. Moreover, genome-wide association studies (GWAS) have identified a large number of pathways and gene sets involved in the development of PDAC (8, 9).

Non-coding RNAs (ncRNAs) have widely been identified in mammals as unique RNA transcripts. Nc-RNAs are classified as small RNAs (<200 bp) and long RNAs (>200 bp) based on nucleotide length, and include microRNAs (miRNAs), PIWI-interacting RNAs (piRNAs), small interfering RNAs (siRNAs), small nucleolar RNAs (snoRNAs), tRNA-derived stress-induced RNAs (tiRNAs), enhancer non-coding RNAs (eRNAs), circular RNAs (circRNAs), and long non-coding RNAs (lncRNAs) (10, 11). In addition, ncRNAs are also categorized based on their localization into cytoplasmic and nuclear ncRNAs. Even though ncRNAs are not translated into proteins, they are critical for DNA replication, translation, RNA splicing and epigenetic regulation. NcRNAs also participate in the cellular processes including differentiation, proliferation, apoptosis and metabolism. Subsequent studies have shown that ncRNAs play a vital role as either oncogenes or tumor suppressors in tumorigenesis. Herein, we summarize the roles and functions of ncRNAs in the diagnosis and treatment of PDAC.

### LncRNAs

**Figure 1:**
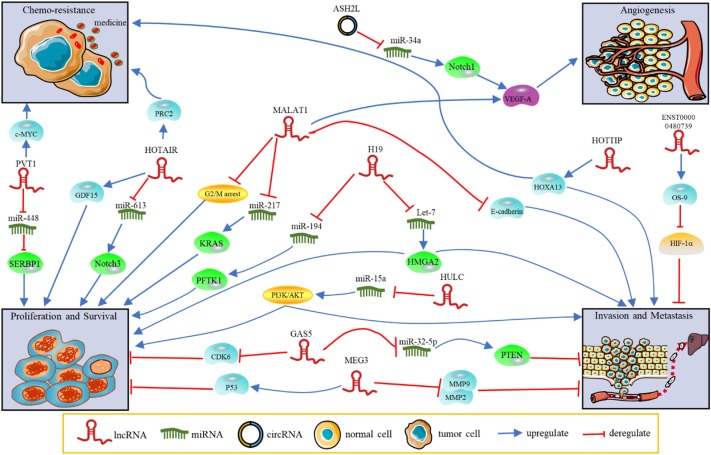
The functions and regulatory mechanisms of ncRNAs in PDAC. NcRNAs regulate tumor progression, such as proliferation, invasion, metastasis, angiogenesis and chemoresistance.

LncRNAs, which contain a length of more than 200 nucleotides, are transcribed by RNA polymerase II and contain a 5′ cap and 3′ poly A tail (12). These ncRNAs are widely distributed throughout the genome but have zero protein-coding capacity. They are involved in many biological processes, including transcriptional regulation in *cis* or *trans*, chromatin remolding, nuclear transport, genomic imprinting and oncogenic progression. Most lncRNAs are expressed in specific different tumor types, making them potential targets for cancer diagnosis and treatment. LncRNAs act as candidate diagnostic biomarkers for PDAC as summarized in Table 1. The functions and regulatory mechanisms of lnRNAs and other ncRNAs are depicted in Figure 1. Therefore, lncRNAs are of interest in the exploration of novel diagnostic and therapeutic approaches.

**Table 1:** LncRNAs as diagnostic biomarkers in PDAC.

	**Source**	**Name**	**Alteration**	**Study sample**	**Sensitivity (%)**	**Specificity (%)**	**Clinicopathological association**	**References**
Single	Serum; serum EV	HULC	Up	PC vs. HC	93.33	96.67	Tumor size, T staging, M staging, and vascular invasion	(13, 14)
			Up	PC vs. BPD	80.00	80.00		(14)
	Serum	HOTAIR	Up	PC vs. HC	-	-	Tumor stage	(15)
	Saliva	HOTAIR	Up	PC vs. HC	78.20	85.60	Unknown	(16)
			Up	PC vs. BPD	80.00	90.00	Unknown	(16)
	Saliva	PVT1	Up	PC vs. HC	96.40	63.60	Unknown	(16)
			Up	PC vs. BPD	69.10	95.00	Unknown	(16)
	Tissue	MALAT1	Up	PC vs. HC	66.00	72.00	Tumor size, clinical stage, lymph node metastasis, distant metastasis	(17)
	Plasma	PINT	Down	PC vs. HC	87.50	77.10	Tumor recurrence	(18)
	Plasma	ABHD11-AS1	Up	PC vs. HC	89.40	88.60	Early pancreatic cancer	(19)
	Plasma	SNHG15	Up	PC vs. HC	68.30	89.60	Tumor differentiation, lymph node metastasis and tumor stage	(20)
Combination (lncRNA)	Saliva	HOTAIR/PVT1	Up	PC vs. HC	78.20	90.90	Unknown	(16)
			Up	PC vs. BPD	81.80	95.00	Unknown	(16)
Combination (lncRNA+CA199)	Plasma	ABHD11-AS1	Up	PC vs. HC	98.50	100.00	Early pancreatic cancer	(19)
	Plasma	PINT	Down	PC vs. HC	85.90	82.90	Tumor recurrence	(18)

*EV, extracellular vesicle; CA199, carbohydrate antigen 199; PC, pancreatic cancer; HC, healthy control; BPD, benign pancreatic disease*.

### LncRNAs as Potential Oncogenes and Biomarkers in PDAC

#### MALAT1

Metastasis-associated lung adenocarcinoma transcript-1 (MALAT1, also known as NEAT2) was initially discovered in lung cancer and has been subsequently detected to be overexpressed in multiple tumors as a negative prognosis factor. MALAT1 is highly expressed in PDAC tissues and positively correlates with tumor size, clinical stage, lymph node metastasis, distant metastasis and prognosis (21, 22). Its expression is also up-regulated in cancer stem cells (CSCs), which is closely related to drug resistance. In addition, it can interact with RNA-binding protein human antigen R (HuR) to regulate T-cell intracellular antigen-1 (*TIA-1*) mediated autophagic activation at the post-transcriptional level. Furthermore, it can also regulate *KRAS* expression through competitive inhibition to promote PDAC cell proliferation (21, 23). Recently, human enhancer of zeste homology 2 (*EZH2*) was shown to be recruited to the E-cadherin promoter through MALAT1, which repressed the expression of E-cadherin and facilitated the invasion and metastasis of PDAC cells (24). Knockout of MALAT1 induced G2/M cell cycle arrest, inhibition of epithelial-mesenchymal transition (EMT), decreased cancer stem cell-like properties, repressed N-myc downregulated gene-1 (*NORG-1*) and hindered the growth and invasion of cancer cells (25, 26). Other work has used different databases to identify the top three key target genes of MALAT1, which include *CCND1*, RAF-mitogen-activated kinase 8 (*MAPK8*) and *VEGFA* (17). This suggests that these may participate in the mTOR signaling pathway, pathways in cancer, and the MAPK signaling pathway in PDAC. Diminished expression of MALAT1 decreases the expression of Yes-associated protein 1 (*YAP1*) and elevates large tumor suppressor 1 (*LATS1*) levels (27). It has been suggested that MALAT1 regulates PDAC via the Hippo-YAP pathway. It also has been reported to regulate multiple signaling pathway, including phosphoinositide-3-kinase-AKT (PI3K-AKT), NF-κb, mTOR, MAPK and WNT pathways in multiple cancer types. Thus, the complex mechanisms and roles that MALAT1 plays in PDAC are worth further exploration.

#### HOTAIR

HOX antisense transcript intergenic RNA (HOTAIR) transcribed from the HOXC locus. Its overexpression has been linked to the poor prognosis of different cancers, including breast, gastric, colorectal, bladder and esophageal squamous cell carcinoma (28–30). Increased expression of HOTAIR has been observed in PDAC tissues and is negatively correlated with overall survival. HOTAIR inhibits the expression of cell cycle interferon related genes, targets and binds to the tumor suppressor gene *GDF15*, and accelerates the proliferation of pancreatic cancer cells. Furthermore, knockout of HOTAIR in the pancreatic cancer cell lines Panc-1 and L3.6Pl significantly decreases the progression of cells and interacts with the Polycomb Repressive Complex 2 (*PRC2*) (28, 31). HOTAIR promotes the proliferation of pancreatic cancer cells by acting as a competing endogenous RNA via sponging miR-613 to regulate the expression of *NOTCH3* (32). Overexpressing HOTAIR regulates the trimethylation of histone H3 at lysine 27 to inhibit the expression of TRAIL receptor death receptor 5 (*DR5*) through *EZH2*. HOTAIR overexpression also improves the resistance of pancreatic cancer cells to TRAIL induced apoptosis (33). In addition, knockout of HOTAIR can enhance the radio-sensitivity of PDAC cells by increasing the expression of Wnt inhibitory factor 1 (*WIF-1*) (34). The HOTAIR-WIF-1 axis can be used as a potential target for PDAC radiotherapy, which needs to be further evaluated. The salivary HOTAIR of pancreatic cancer patients was significantly higher than expression levels observed in healthy individuals. The expression of HOTAIR in patients' saliva was significantly reduced after the PDAC tumor was surgically removed (16). This indicates that HOTAIR can be evaluated in patients undergoing resection and that it may be a promising novel diagnostic marker and therapeutic target.

#### HOTTIP

The lincRNA HOXA distal transcript antisense RNA (HOTTIP) is another HOX-related lncRNA. Studies have found that the expression levels of HOTTIP are significantly increased in multiple PDAC cell lines and PDAC specimens (35). HOTTIP interacts with the WD repeat containing protein 5 (*WDR5*)/mixed lineage leukemia (*MLL*) complex to enhance the methylation of histone 3 on lysine 4 (*H3K4*) in order to modulate the proliferation and differentiation of PDAC cells (36, 37). Up-regulation of HOTTIP promotes the secretion of IL-6 and expression of *PD-L1* in neutrophils, thereby inhibiting the activity of T cells and promoting the immune escape of ovarian cancer cells (38). This may be used for reference in immunotherapy of PDAC. Decreased expression of HOTTIP in pancreatic cancer cells leads to increased G0/G1 phase cells, decreased Vimentin and Snai1 expression, and increased E-cadherin expression. Furthermore, HOTTIP knockout can reduce the expression level of *HOXA13* and enhance the sensitivity of human pancreatic cancer cells to gemcitabine (35). In turn, others have shown that HOTTIP is not involved in the regulation of *HOXA13*, but regulates several other HOX genes (39). Further studies will be required to fully understand these relationships.

#### PVT1

Plasmacytoma variant translocation 1(PVT1) was the first lncRNA gene identified in human Burkitt's lymphoma as a recurrent breakpoint. PVT1 and *MYC* have correlated one another and co-amplified. It was confirmed in a variety of solid tumors, including colon and breast cancers, that increased expression of PVT1 could increase *MYC* protein (40). A GWAS study identified a risk locus at 8q24.21, which interacts with *MYC* promoters, that reached genome-wide significance located PVT1 (41). It was also identified that in human PDAC cells, PVT1 acts as an oncogene promoting EMT via TGF-β/Smad signaling (42). PVT1 also acts as a sponge for miRNAs to regulate the development of PDAC. PVT1 could promote the proliferation and metastasis of PDAC cells by acting as a miR-448 sponge to inhibit *SERBP1* (43). It has also been reported that PVT1 acts as a sponge to modulate cytoprotective autophagy and promote the development of PDAC via the PVT1/miR-20a-5p/ULK1/autophagy pathway (44). Of note, PVT1 also participates in drug resistance. Research indicates that PVT1 regulates gemcitabine chemosensitivity in PDAC through miR1207 (45). Curcumin can inhibit the PRC2-PVT1-c-Myc axis by inhibiting the PRC2 subunit Enhancer of *EZH2* to enhance the sensitivity of PDAC cells to gemcitabine (46). PVT1, along with MALAT1 and HOTTIP, could act as a prospective biomarker to predict the efficacy of gemcitabine in PDAC patients (47), and as such, future assessment is warranted.

#### H19

LncRNA H19 is a maternally imprinted gene that is highly expressed in PDAC tissues and is involved in tumor progression. It increases high-mobility group AT-hook 2 (*HMGA2*) mediated EMT by antagonizing let-7 and promotes both tumor cell metastasis and invasion (48, 49). MiR-675 reduces the activation of H19 by binding to the 3′ untranslated region (UTR) on *E2F-1* mRNA and altering the expression of *E2F-1* protein (50). In addition, the Wnt-signaling pathway is involved in regulating PDAC cell proliferation and migration via the H19/miR-194/PFFTK1 axis (51). H19 is also believed to play a role in cancer therapy and is the earliest lncRNA used in the treatment of PDAC. BC-819 (DTA-H19) carries a diphtheria toxin-A chain (DTA), which can be applied to the treatment of tumors expressing high levels of H19 (52). DTA-H19 combined with gemcitabine can reduce the tumor size and delay the progression of PDAC *in vivo*. Altogether, these data suggest that H19 is a promising therapeutic marker for PDAC.

#### HULC

Highly up-regulated in liver cancer (HULC) is another lncRNA that modulates the proliferation of PDAC. HULC can regulate the viability, proliferation, migration and invasion of PDAC cells. Up-regulation of HULC activates the PI3K/AKT pathway via negative regulation of miR-15a expression (53). HULC levels are significantly increased in PDAC compared to the non-tumor tissues. Higher expression of HULC in PDAC is correlated with poor clinical outcomes in patients. It is thus suggested that HULC is a promising prognostic biomarker candidate (54). Recently, a report suggested that serum extracellular vesicle (EV) HULC expression is increased in PDAC patients in comparison to intraductal papillary mucinous patients and healthy individuals (13). Thus, HULC may be a new potential diagnosis maker for PDAC and may merit further investigation.

### LncRNAs as Potential Suppressors and Biomarkers in PDAC

#### GAS5

Growth arrest-specific transcript 5 (GAS5) was originally identified by screening potential tumor suppressor genes expressed at high levels during growth using a functional cDNA library (55). GAS5 is one of the few lncRNAs that are negatively correlated with tumor development in breast cancer, malignant pleural mesothelioma and hepatocellular carcinoma (56–58). GAS5 negatively regulates miR-32-5p to promote the expression of pleiotrophin (*PTEN*), which can block the activation of the PI3K/Akt signaling pathway, inhibiting the proliferation and survival of PDAC cells (59). Studies have found that GAS5 overexpression can significantly inhibit both the proliferation and invasion of PANC-l and BxPC-3 cells *in vitro* (60). After inhibiting of GAS5 expression by RNA interference, a larger number of cells were found to be arrested in the S phase of the cell cycle. This suggested that GAS5 regulates the cell cycle of PDAC. Furthermore, GAS5 regulates the cell cycle of PDAC cells by inhibiting cyclin-dependent kinase 6 (*CDK6*) and blocking proliferation and differentiation. Studies have shown that GAS5 inhibits drug resistance in PDAC by negative regulation of miR-181c-5p and reducing the inactivation of the Hippo signal transduction pathway (61). Overexpression of GAS5 inhibits PDAC cell proliferation, migration and gemcitabine resistance through miR-221/suppressor of cytokine signaling 3 (*SOCS3*) mediated EMT and tumor CSCs (62). Overall, GAS5 could be as a novel target for PDAC drug resistance therapy.

#### LncRNA ENST00000480739

The lncRNA ENST00000480739 is a relatively rare tumor suppressor that was recently uncovered. ENST00000480739 expression in pancreatic tumor specimens is significantly lower than that which is observed in adjacent non-tumor tissues (63). It is also negatively correlated to tumor stage and could be used as an independent prognostic factor in PDAC patients who underwent surgery. ENST00000480739 inhibits tumor invasion through the regulation of osteosarcoma amplified-9 (*OS-9*), modulates hypoxia-inducible factor-1α (*HIF-1*α) and inhibits EMT (63). It not only has the potential to inhibit metastasis but can also be used as a biomarker for both risk prediction and treatment screening in PDAC.

#### MEG3

Maternally expressed gene 3 (MEG3) acts as a tumor suppressor and shows to be down-regulated in several tumors, such as hepatocellular, prostate, gastric and lung cancers (64–67). Hu et al. (68) found that MEG3 inhibits the proliferation, induces apoptosis via p53 activation and is upregulated along with p53 by fenofibrate to restrain the proliferation of PDAC cells. Other studies found that the MEG3 expression levels in human pancreatic cancer tissues are lower than corresponding non-cancerous tissues (69). These were also found to be negatively correlated to patients' clinicopathological features. *In vitro* studies have shown that MEG3 plays an anticancer role in the regulation of cell proliferation, migration, invasion, induction of EMT, and cancer stem cell (CSC) properties. Furthermore, study has shown that MEG3 overexpression plays an anticancer role through the *in vitro* modulation of the PI3K/AKT/ B cell lymphoma-2 (Bcl-2)/Bax/Cyclin D1/P53 and P13K/AKT/matrix metalloproteinases-2(MMP-2) /MMP-9 signaling pathways (70).

Numerous lncRNAs participate in pancreatic cancer tumorigenesis. Further, many of the PDAC susceptibility loci that were previously identified in GWAS are located in lncRNAs such as 7q32.3 (LINC-PINT) and 17q25.1 (LINC00673) (41, 71). Interestingly, LINC-PINT, through the TGF -β pathway, inhibits PDAC growth in early stages (72). This may be a potential target for early treatment for patients but it requires further testing to prove. Additionally, LINC00673 is also correlated with good outcomes in PDAC patients. It can negatively regulate miR-504 to inhibit the progression of PDAC (73). There are many potential lncRNAs biomarkers that remain to be explored and translated to clinical practices.

### CircRNAs

Circular RNAs (circRNAs) are a new type of endogenous ncRNA that used to be considered as miRNA sponges. CircRNAs are stable since they lack 5′ cap or 3′ Poly A tail terminal ends that block traditional RNA degradation pathways, existing as a closed loop structure. Heterogeneous circRNAs may contribute to the development of many different tumors.

### CircRNAs in PDAC

Numerous studies have demonstrated that circRNAs are aberrantly expressed in PDAC. One study identified circRNAs in six pairs of PDAC and para-cancerous tissues using a microarray (74). Additional microarray data revealed that there were 115 upregulated and 141 downregulated circRNAs in PDACs (75). Another study uncovered that 453 circRNAs were differentially expressed and were significantly different in extracellular vesicles isolated from the plasma of 8 PDAC patients or healthy controls (76). It has been suggested that aberrant expression of circRNAs in PDAC are related to proliferation and development. These aberrantly expressed circRNAs may be involved in the regulation of PDAC and are expected to be diagnostic markers, though this remains to be tested.

### CircRNAs Regulate PDAC Progression

CircRNAs can regulate a variety of different pathways (Table 2). The circRNA ciRS7 is expressed in PDAC tissues compared to para-cancerous tissues and can negatively regulate miR-7, a cancer suppressor. It can also affect the proliferation and invasion of PDAC cells through epidermal growth factor receptor (*EGFR*), as well as signal transducer and activator of transcription 3 (*STAT3*) signaling pathways (77). In addition, overexpression of ciRS7 promotes lymph node metastasis and venous invasion in PDAC cases. Down-regulation of circ-LDLRAD3 can inhibit PDAC cell proliferation and metastasis through the up-regulation of miR-137-3p/ PTEN (80). Others have proven that the circRHOT1 regulates the PDAC cells proliferation, invasion and metastasis by binding to miRNAs, including miR-26b, miR-125a, miR-330, and miR-382 (83). There is mounting evidence underlining that circRNAs act as miRNA sponges that regulate the progression of PDAC. Chen et al. (79) elucidated that silencing of circRNA_100782 down-regulates the expression levels of interleukin-6 receptor (*IL6R*) and *STAT3* by acting as a miR-124 sponge that inhibits BxPC3 cell proliferation. Another study has shown that circ_0030235 promotes cell proliferation by directly sponging miR-1253 and miR-1294 *in vitro* (78). An additional study also revealed that circ_0007534 functions as a sponge for miR-625 and miR-892b to facilitate the malignant behavior of PDAC cells (82). Studies have identified that circZMYM2 (hsa_circ_0099999) is up-regulated in both PDAC cells and tissues, promoting tumor progression by influencing *JMJD2C* expression levels via acting as miR-355-5p sponges (84).

**Table 2:** Function of circRNAs in PDAC.

**circRNAs**	**Alteration**	**Function**	**Targeted miRNA**	**Involved genes/pathways**	**References**
ciRS7	Up	Promote invasion and metastasis	miR-7	EGFR/STAT3 signaling pathway	(77)
circ_0030235	Up	Promote tumor progression; prognostic marker	miR-1253 and miR-1294	-	(78)
circRNA_100782	Up	Promote cell proliferation	miR-124	IL6-STAT3 pathway	(79)
circ-LDLRAD3	Up	Promote tumor invasion, and metastasis; diagnosis biomarkers	miR-137-3p	*PTN*	(80, 81)
circ_0007534	Up	Promote tumor progression; diagnosis and prognostic factor	miR-625 and miR-892b	-	(82)
circRHOT1	Up	Promote tumor cell proliferation, invasion, and metastasis	miR-26b, miR-125a, miR-330 and miR-382	-	(83)
circZMYM2	Up	Promote proliferation and invasion, and inhibit apoptosis	miR-355-5p	*JMJD2C*	(84)
circ-PDE8A	Up	Promote tumor progression; prognostic factor	miR-338	MACC/MET/ERK pathway	(85)
circ-IARS	Up	Promote metastasis; prognostic factor	miR-122	-	(86)
circ-ASH2L	Up	Promote tumor invasion, proliferation and angiogenesis	miR-34a	-	(87)
chr14:101402109-101464448+, chr4:52729603-52780244+	Up	Chemo-resistant	Unknown	Unknown	(88)
has_circ_0001649	Down	Inhibit cell proliferation, prognostic factor	Unknown	Unknown	(89)

### CircRNAs as Diagnostic, Prognostic and Therapeutic Biomarkers for PDAC

CircRNAs have the ability to be strong biomarkers for PDAC since they exhibit stable expression and have high serum concentrations. Yang et al. (81) investigated that circ-LDLRAD3 up-regulation in PDAC tissues, plasmas and PDAC cell lines, and identified its association with venous and lymphatic invasion and metastasis. Furthermore, this study confirmed that circ-LDLRAD3 holds potential to be a new diagnosis biomarker, as it has higher sensitivity and specificity when combined with CA19-9 than using CA19-9 alone (81). Clinical studies have shown that the overexpression of circ_0030235, circ_0007534 in PDAC are associated with tumor stage, lymph node invasion and poor overall survival (78, 82). Exosomes play an important role in the development and prognosis of tumors. Owing to their stability, they can be detected in blood plasma and equipped higher level than existing checks. Studies have identified that exosomal circ-PDE8A promotes the progression of PDAC by binding to miR-338 to activate the MACC/MET/ERK pathway (85). The same group also found that high expression levels of circ-IARS in plasma exosomes positively correlated with tumor metastasis including vessel invasion, liver metastasis and tumor-node-metastasis (TNM) stage. circ-IARS is also negatively associated with survival of PDAC patients (86). Shao et al. (88) developed Gemcitabine resistant cell lines (PANC-1-GR) and verified that the expression levels of two circRNAs (chr14:101402109-101464448+, chr4:52729603-52780244+) were significantly correlated to drug resistance observed in PANC-1-GR, as well as the plasma of gemcitabine non-responsive PDAC patients. CircRNAs can be used not only as biomarkers, but also to provide information about tumor stage and classification. Conversely, expression levels of has_circ_0001649 were decreased in PDAC tissues and cells when compared to normal control, and were associated with tumor stage and differentiation grade (89). Has_circ_0001649 may be regarded as a novel prognostic biomarker for PDAC patients who had undergone surgery.

Currently, the roles of circRNAs in tumor progression and clinical application have gained attention. However, the studies of circRNAs in PDAC are still at infancy stage. Further studies on circRNAs in PDAC should not only be based on the databases established, but also require a great deal of work to fully understand their functions.

### MiRNAs

MicroRNAs (miRNAs) are small non-coding RNAs ~19–25 nucleotides in length, that regulate gene expression at the post transcriptional level through RNA interference (90). MiRNAs play a significant role in the initiation and progression of a tumor by regulating tumor growth, anti-apoptotic, metastasis and invasion (91, 92). These have potential as cancer diagnosis markers, prognosis predictors, and for the monitoring of therapy.

### MiRNAs as Diagnosis and Prognostic Biomarkers for PDAC

MiRNAs are conservative, generalized, testable and keep stable when existing outside cells. Ongoing research has revealed that miRNAs exhibit fair sensitivity and specificity as diagnosis biomarkers. The potential for miRNAs as diagnostic and therapeutic markers leads to the analyses of deregulated miRNAs in PDAC cases (93). Abnormal expression levels of miRNAs in PDAC tissues, blood and saliva have shown to be closely related to the initiation and development of PDAC (94, 95). Studies have found that plasma miRNAs (miR-16, miR-196a) combined with serum CA19-9 can increase the sensitivity and specificity for diagnosis in comparison with using CA19-9 alone (96). MiRNAs can also be identified in the saliva and act as diagnosis biomarkers for unresectable pancreatic tumors (95). This can lead to the creation of specific miRNA profiles for diagnosis and treatment. In addition, published studies have demonstrated that miRNAs can interact with lncRNAs and circRNAs (Figure 1). They can also regulate key signaling molecules and pathways in disease development and progression (Tables 2, 3). At the same time, it is challenging to fully understand the interaction network between different miRNAs and other ncRNAs. Further differentiation of the roles that miRNAs play in the development and progression of PDAC are necessary to explore their specificity and sensitivity as biomarkers for PDAC.

**Table 3:** Function of up/down-regulated miRNAs in PDAC.

**miRNAs**	**Alteration**	**Function**	**Confirmed targets**	**References**
miR-21	Up	Promote the cell growth, invasion and migration; prognostic factor, Chemo-resistant	*PI3K/AKT/PTEN, PDCD4, BCL-2, FASL, TGF-β1, P85α, VHL*	(97–101)
miR-155	Up	Promote tumor progression, invasive and migration, mediate apoptosis; therapeutic and prognostic factor	*TP53INP1, SOCS1, SOCS3*	(102–104)
miR-221/222	Up	Promote tumor progression, proliferation and invasion, inhibit apoptosis; Chemo-resistant	*MMP-2, MMP-9, TIMP-2, PTEN, *P*27^**kip*1*^, *P*57^**kip*2*^, PUMA*	(105–107)
miR-320a	Up	Promote progression, Chemo-resistant	*PDCD4*	(108)
miR-10a	Up	Promote proliferation, invasion and metastatic; Chemo-resistant, therapeutic and prognosis factor	*HOXA1, HOXA3, TFAP2C*	(109–111)
miR-31	Up	Promote tumor development and progression, promote invasion and migration; Chemo-resistant, prognostic factor	*RASA1, ARID1A*, Pancreatic stellate cells	(112–114)
miR-210	Up	Promote migration; diagnosis and prognostic factor	Pancreatic stellate cells	(115, 116)
miR-196b	Up	Promote tumor progression and invasion; diagnosis and prognostic factor	*CADM1*	(117–119)
miR-23a	Up	Promote tumor progression, promote cell proliferation, invasion, inhibit apoptosis; prognostic factor	*ESRP1, APAF1, FOXP2*	(120–122)
miR-451	Up	Promote cell proliferation and metastasis	*CAB39*	(123)
miR-34a	Down	Inhibit cell growth, migration, invasion, progression, induce apoptosis; diagnosis, therapeutic and prognostic factor	*CCND1, E2F1, E2F3, BCL-2, C-MYC, SNAIL1, CDK6, SIRT1, NOTCH1/2/4, SMAD3*	(124–128)
miR-100	Down	Inhibit tumor cell proliferation; increase drug sensitivity	*FGFR3*	(129)
miR-217	Down	Inhibit cell growth, invasion, induce apoptosis; diagnosis and prognostic factor	*KRAS, E2F3, TPD512, SIRT1*	(130–132)
miR-143	Down	Inhibit tumorigenesis, inhibit tumor cell migration, invasion, metastasis and xenograft	*KRAS, RREB1, GEF1, GEF2, COX2, TAK1*	(133–136)
miR-141	Down	Inhibit cell proliferation, invasion, migration and metastasis, induce apoptosis; prognostic factor	*YAP1, WIPF1, TM4SF1, MAP4K4, NRP-1*	(137–141)
miR-200	Down	Inhibit metastasis; Chemo-resistant, prognostic factor	*PTEN, MT1-MMP, ZEB1, ZEB2, SOX2*	(142–145)
miR-375	Down	Inhibit cell growth; prognostic factor	*PDK1, ZFP36L2, IGFBP5, CAV1*	(146–148)
miR-148a	Down	Inhibit cell proliferation, migration and invasion, promote apoptosis; diagnosis, prognostic factor	*CCKBR, BCL-2, PHLAD2, CDC25B, WNT10b, ERBB3, AMPKα1, DNMT1*	(149–153)
miR-let7	Down	Inhibit cell growth, proliferation; Chemo-resistant, therapeutic and prognostic factor	*HMGA1, HMGA2, IGF2BP1, IGF2BP3, KRAS, SOCS3, RRM2, N-cadherin/ZEB1*	(154–157)
miR-216	Down	Inhibit cell growth, promote apoptosis; therapeutic factor	*JAK2, BECLIN-1*	(158, 159)
miR-146a	Down	Inhibit cell invasion and metastasis	*EGFR, MTA-2, IRAK1*	(160)

*TM4SF1, transmembrane-4-L-6-family-1; MAP4K4, mitogen-activated protein kinase isoform 4; TIMP-2, tissue inhibitor of metalloproteinases-2;VHL, Von Hippel-Lindau tumor suppressor; SOCS, suppressors of cytokine signaling; FGFR3, fibroblast growth factor receptor 3; RREB1, Ras-responsive element-binding protein; GEFs, guanine nucleotide exchange factors; COX, cyclooxygenase; TAK1, TGF-β-activating kinase 1; TFAP2C, transcription factor activating protein 2 gamma; ARID1A, AT-rich interactive domain 1A; ESRP1, epithelial splicing regulatory protein 1; APAF1, apoptotic protease activating factor 1; FOX, forkhead box; CAB39, calcium-binding protein 39; NRP-1, neuropilin-1; MT1-MMP, membrane type-1 matrix metalloproteinase; IRAK1, interleukin 1 receptor-associated kinase 1; CCKBR, cholecystokinin-B receptor; JAK2, Janus kinase 2;;IGF2BPs, insulin growth factor 2 binding proteins*.

## Conclusion

With the rocket development of next-generation sequencing and bioinformatic analyses, ncRNAs and their prominent roles in oncogenesis, specifically the progression of PDAC reveal enormous potential for ncRNAs in the diagnosis and treatment of cancers. Although ncRNAs have gradually become a research hotspot, the limitations in detection approaches and inclusion in larger databases make their roles in PDAC difficult to fully understand. Understanding the relationship between the function and mechanism of ncRNAs in PDAC will help classify ncRNAs and their roles in the clinic. A great deal of work remains to be completed to uncover the complex mechanisms of ncRNAs, which lead to tumorigenesis and progression, to ultimately select the most effective diagnostic and therapeutic targets.

## Author Contributions

RG and YJ designed the study. RG collected data and wrote the manuscript. YJ revised the manuscript. All authors read and approved the final manuscript.

### Conflict of Interest

The authors declare that the research was conducted in the absence of any commercial or financial relationships that could be construed as a potential conflict of interest.
